# *In Vivo* and *In Vitro* Toxicity Profiles of Hexane Extract of *Alpinia malaccensis* Rhizome in Rat and Cell Line Models

**DOI:** 10.1155/2021/9578474

**Published:** 2021-01-17

**Authors:** T. Somarathna, M. G. Thammitiyagodage, K. K. D. S. Ranaweera, G. A. S. Premakumara, M. A. Akbarsha, B. Kadalmani, N. S. Weerakkody

**Affiliations:** ^1^Department of Agricultural & Plantation Engineering, Faculty of Engineering Technology, The Open University of Sri Lanka, Nawala, Sri Lanka; ^2^Animal Centre, Medical Research Institute, No: 527, Dr Danister de Silva Mawatha, Borella, Sri Lanka; ^3^Department of Food Science and Technology, Faculty of Applied Sciences, University of Sri Jayewardenepura, Nugegoda, Sri Lanka; ^4^Deapartment of Basic Sciences & Social Science, Faculty of Nursing, University of Colombo, Colombo 3, Sri Lanka; ^5^Mahatma Gandhi-Doerenkamp Center, Bharathidasan University, Tiruchirappalli, India; ^6^Departmentof Biotechnology & Research Co-ordinator, National College (Autonomous), Tiruchirappalli 620001, India; ^7^Department of Animal Science, School of Life Sciences, Bharathidasan University, Tiruchirappalli, India; ^8^UGC National Centre for Alternatives to Animal Experiments, Bharathidasan University, Tiruchirappalli, India

## Abstract

The objective of the study was to evaluate the potential toxicity of crude *n*-hexane extract of *Alpinia malaccensis* rhizome. The *in vivo* acute oral toxicity was evaluated by administering a single oral dose of the extract at 0, 300, or 2000 mg/kg body weight to female Wistar rats according to modified OECD Test Guideline 423. For the *in vitro* cytotoxicity study, A549, HepG2, 3T3, and COS-7 cell lines were exposed to different doses of *A. malaccensis* extract and cell viability was assessed adopting MTT assay followed by AO/EB staining, Hoechst staining, and comet assay with a view to compare the cellular and molecular mechanisms underlying the toxicity, if any. It was found that administration of 2000 mg/kg bw dose in *in vivo* oral acute toxicity study did not produce significant toxicity or mortality. No significant (*p* < 0.05) differences were observed for body weight and hematological and biochemical parameters compared to control after 14 days of treatment. No changes in behavior, body weight, hematological and biochemical parameters, and aspects of histopathology were observed when compared to the control. Thus, the possible oral lethal dose for *A. malaccensis* extract is above 2000 mg/kg body weight. The *in vitro* cytotoxicity analysis showed nontoxicity concentrations of the extract to be 2, 1.4, 30, and 1.4 *µ*g/mL for A549, HepG2, 3T3, and COS-7 cells, respectively, where no apoptotic/necrotic cell death and DNA damage were observed. In conclusion, the extract of rhizome of *A. malaccensis* did not produce apparent cytotoxicity or acute oral toxicity, confirming the scope to use *A. malaccensis* as a safe food preservative and a natural therapeutic product after further subacute and chronic toxicity studies.

## 1. Introduction

Medicinal plants have been widely used in conventional or traditional medicinal practices as a reliable cure to diseases since prehistoric days. The World Health Organization also has recognized plants as a reliable source of therapeutics [1]. The therapeutic property of the medicinal plants lies in the bioactive compounds they contain. It is believed that the plant-derived compounds, when used as therapeutics, are less harmful compared to the synthetic drugs [2]. Thus, the medicinal plants offer great scopes to discover newer drugs. Nevertheless, surveys have indicated that many plants used in traditional medicines can potentially produce adverse effects [3, 4], and some of them have been shown to contain toxic compounds [5]. Therefore, not all medicinal plants are safe. Hence, there is pertinent need to assess the toxicity of medicinal plants to ensure safety.

*Alpinia malaccensis,* popularly known as Malacca ginger and “Rankihiriya,” belongs to the family Zingiberaceae. It is a perennial South Asian medicinal plant cultivated widely in the tropical regions of Asia including Indo-China, Bangladesh, and Sri Lanka [6]. *A. malaccensis* has traditionally been used in the preparation of medicines to cure nausea, vomiting, and wounds and also as a seasoning ingredient in the processing of meat [7]. Somarathna et al. [8] evaluated the bioactive chemical constituents of the hexane crude extract of *A. malaccensis* rhizome and found 82.87% 1'-acetoxychavicol acetate (1'ACA) as the major active chemical compound. Crude *A. malaccensis* extract as well as the pure compound 1'ACA showed strong antibacterial and anti-biofilm activities against *Listeria monocytogenes* and *Staphylococcus aureus* [8, 9].

However, Primahana et al. [10] reported that trans-methyl cinnamate found in *A. malaccensis* produces moderate toxicity to brine shrimp at 120.47 *µ*g/mL LC_50_. Further, Thu et al. [11] reported that fruit of *A. malaccensis* induces cytotoxicity in A549 and HepG2 cell lines at 4.8 and 5.5 *µ*g/mL doses, respectively. It was suggested that the inconsistency of the chemical composition may be due to the differences in the plant parts used, age, variety, edaphic factors, and geographical regions where cultivated [12, 13]. Also, Chan et al. [14] reported that the rhizome accumulates more secondary chemicals than the other plant parts. Therefore, evaluation of toxicological aspects of *A. malaccensis* rhizome will be a rewarding area of research.

The present study was aimed at investigating the potential toxicity of *n*-hexane extract, which would contain the most nonpolar compounds, of rhizome of *A. malaccensis* by finding the acute *in vivo* toxicity using the rat model and *in vitro* cytotoxicity using cell lines.

## 2. Materials and Methods

### 2.1. Plant Collection and Authentication

*A. malaccensis* plants were collected from the medicinal garden of the Nature Secret (Pvt) Ltd, Millewa, Horana, Sri Lanka, and a voucher specimen (2012/APE/02)was deposited in the herbarium of Department of Agricultural Plantation Engineering, the Open University of Sri Lanka. The plant was identified using the key to species by Dassanayake and Forsberg [15].

### 2.2. Extraction of *A. malaccensis*

Fresh *A. malaccensis* rhizomes were washed in running water, and the outer skin was peeled off. The rhizome was sliced and oven-dried at 40°C for 24 h. The dried slices were pulverized using a grinder (National Super Blender, Taiwan, Model MX-TIIOPN) for 1 minute at 30 seconds interval in each cycle for five times. The powder was stored at −20°C until use. *n*-Hexane was used as the solvent for the extraction. The extract was prepared by adding 10 g of *A. malaccensis* powder to 100 mL of *n*-hexane and agitated (160 rpm) for 24 h at 30°C in a rotary shaker (Stuart® orbital shaker SSL1, UK). The mixture was centrifuged at 4500 ×g for 10 min (Centurion Scientific Ltd, UK), and the supernatant was filtered using #1 Whatman filter paper. The filtrate was evaporated under vacuum at 40°C using a rotary evaporator (KIA RV 5, Switzerland) and filter-sterilized through a 0.45 *µ*m filter unit (Millex® HA, Germany). The filtrate was N_2_-fluxed under heat (40°C) for 3 h until a hexane-free extract was obtained. Finally, the concentrated extract was stored at 4°C until use.

### 2.3. Cell Culture

Human lung carcinoma cell (A549), hepatocarcinoma cell (HepG2), normal mouse fibroblast cell (3T3), and monkey kidney tissue cell (COS-7) were obtained from the National Center for Cell Science (NCCS), Pune, India. The cells were maintained in DMEM medium supplemented with 10% fetal bovine serum (FBS, Invitrogen, USA) and with 20 mL each of penicillin and streptomycin as antibiotics, in a humidified atmosphere of 5% CO_2_ and 95% air, in a CO_2_ incubator (Thermo Scientific, USA) [16]. The cytotoxicity studies were conducted at Mahatma Gandhi-Doerenkamp Center for Alternatives at Bharathidasan University, Tiruchirappalli, India.

### 2.4. MTT Assay for Assessment of Cell Viability

Cell viability, which would reveal the cytotoxic property of the extract, was evaluated using the 3-(4, 5-dimethylthiazol-2-yl)-2, 5-diphenyltetrazolium bromide (MTT) colorimetric assay with slight modifications [17]. Cells were seeded in 96-well plates at 5 × 10^3^ cells/well and incubated for 24 h at 37°C. The cells were treated with the extract at increasing concentrations within 0–1000 *µ*g/mL for 24 h, at 37°C. The extract was quantitatively dissolved in minimum quantity of DMSO and diluted in the culture medium to prepare the stock solution, which was then made up in the culture medium so as to have the final extract at DMSO concentration of 0.1%. This concentration of DMSO is known not to affect the cell viability [18]. DMSO at this concentration was also used as the solvent control. Experiments with each extract concentration were conducted in triplicates on the same batch of cells. After 24 h incubation, 20 mL of MTT (Sigma-Aldrich, St. Louis, MO, USA) solution (5 mg/mL in PBS) was added to each well and incubated for 3 h at 37°C. The medium was then removed, and 100 mL of DMSO was added to each well to dissolve the purple formazan product. The absorbance was measured at 570 nm (measurement) and 630 nm (reference) using a 96-well plate reader (Bio-Rad, Hercules, CA, USA). The percentage inhibition was calculated from these data using the following formula, and IC_50_, defined as concentration of the test substance at which cell viability is decreased to 50%, was calculated.

(1) $$\begin{array}{c} \text{Percentage of cell inhibition}=\frac{\text{mean OD }\left(\text{control}\right)-\text{mean OD }\left(\text{treatment}\right)}{\text{mean OD }\left(\text{control}\right)}x\;100. \end{array}$$

### 2.5. Morphological Assessment of Cell Death Using AO/EB Fluorescent Assay

The morphological characteristics of cells in respect of apoptosis and necrosis were assessed by AO/EB staining [19]. The A549, HepG2, 3T3, and COS-7 cells were cultured in 6 well plates and incubated for 24 h with IC_50_ and no toxicity concentrations of the extract, as found in the MTT assay. The treated and untreated cells were centrifuged (3000 rpm for 4 min) and incubated with acridine orange (AO) and ethidium bromide (EB) solutions (1 part of 100 mg/mL each of AO and EB in PBS) and observed in a fluorescent microscope (Carl Zeiss, Jena, Germany) using a UV filter (450–490 nm). Three hundred cells per sample were counted, in duplicate, and scored as viable or dead, and if dead whether by apoptosis or necrosis as judged from nuclear morphology and cytoplasmic organization. Then, the percentages of apoptotic and necrotic cells were calculated. The morphological features of interest were photographed.

### 2.6. Assessment of Nuclear Morphological Features Using Hoechst 33528 Staining

The nuclear morphological features of the cells were assessed using Hoechst 33528 staining [20]. The cells were cultured in 6-well plates and treated with *A. malaccensis* at its IC_50_ concentration and no toxicity concentration, for 24 h. After incubation, the treated and control cells were harvested and stained with Hoechst 33258 (1 mg/mL in PBS) for 5 min at room temperature. A drop of cell suspension was placed on a glass slide and covered with a cover slip. Three hundred cells, each in triplicate, were observed at x400 in the fluorescent microscope fitted with a 377–355 nm filter. The percentage of cells reflecting pathological changes was calculated.

### 2.7. Genotoxicity Assessment Using Comet Assay

Genotoxicity assessment was performed using comet assay where single-cell gel electrophoresis was performed to quantify the DNA damage [21]. The cells were treated with the IC_50_ concentration and no toxicity concentration of the extract for 24 h. The harvested cells were suspended in low melting point agarose in PBS and pipetted out to microscope slides on precoated layer of normal melting point agarose. Another layer of medium melting agarose was laid on top of the low melting agarose. Slides were immersed in prechilled lysis buffer (2.5 M NaCl, 100 mM Na_2_EDTA, 10 mM Tris, 0.2mMNaOH (pH 10), and Triton X-100) and incubated overnight at 4°C in order to lyse the cells and allow DNA unwinding. Then, the slides were exposed to alkaline buffer (300 mM NaOH, and 1 mM Na_2_-EDTA (pH > 13)) for 20 min to allow DNA unwinding and then subjected to electrophoresis at 7 V. The slides were washed with neutralization buffer (0.4 M Tris, pH 7.5) for 2 min and observed in the fluorescent microscope. The triplicate data, each from one hundred and fifty cells, from each treatment group, were collected manually. The images were used to evaluate the degree of DNA damage representing the fraction of total DNA in the tail, according to Gayathri et al. [16].

### 2.8. Acute Oral Toxicity Study Using Wistar Rats

#### 2.8.1. Animals

The acute oral toxicity test was carried out using female Wistar rats. The number of animals and sex used for each test were determined considering both Organization of Economic Cooperation and Development (OECD) guidelines and the opinion of the Ethics Review Committee of Medical Research Institute (2016/22), Sri Lanka, based on the 3R concept. All animals were maintained under standard laboratory conditions including 20°C to 24°C temperature, 50% to 70% relative humidity, and a light regimen of 12 h light and dark throughout the experimental period. Standard size, polycarbonate, and transparent cages were used for animal housing. Three rats from the same sex were kept in one cage. Sterilized wood shavings were used as the bedding material. All animals were fed with MRI rabbit and rat formula prepared according to WHO guideline given by Saboudry [22]. The formulations were prepared at the Medical Research Institute using locally available ingredients. All rats were acclimatized to laboratory conditions for a period of 7 days prior to dosing.

#### 2.8.2. Acute Oral Toxicity Test

Acute oral toxicity test of the crude *n*-hexane extract of rhizome of *A. malaccensis* was carried out according to the method described in OECD Test Guideline 423. Three-month-old female Wistar rats, weighing 150–200 g, were randomly divided into three groups, control (*n* = 3) and two treatment groups (*n* = 3 each). The animals were maintained in an air-conditioned and light-controlled room with access to water and feed *ad libitum* throughout the experiment. Animals were kept fasting overnight with free access to water the day prior to starting the experiment. Animals were individually weighed. Rats in the control group were administered olive oil (1 mL). Doses of 300 and 2000 mg/kg BW of crude extract were used for the limit test. The administered volume was adjusted to 1 mL/kg BW per rat.

After administration of *A. malaccensis* extract, rats were observed for first 30 min, and first 24 h with special attention during first 4 h, and thereafter daily for 14 days. The rats were weighed and observed for signs of toxicity including mortality, changes in behavioral pattern (salivation, lethargy, and sleep), physical appearance, injury, pain, and signs of illness during the observation period. In addition, the body weight, feed intake, and water consumption were recorded throughout the experimental period. Animals were mildly sedated using gaseous anesthesia, and 1 mL of blood was collected by the lateral tail vein puncture technique for biochemical and hematological analysis. The serum creatinine, alanine aminotransferase (ALT), aspartate aminotransferase (AST), and blood urea nitrogen (BUN) were measured with commercially available estimation kits (Pointe Scientific, Colombo) using a semiautomated biomedical analyzer (Stat Fax 3300, Ramsey, MN, USA). At the end of the experiment, the rats were humanely euthanized by overdosing gaseous anesthesia, and the liver, kidney, lung, heart, and spleen were collected. The relative organ weights of rats were recorded and also examined macroscopically. The organs were preserved in a fixation medium of 10% solution of formalin for histopathological study.

### 2.9. Histopathological Study

Portions of formalin-fixed liver, kidney, lung, heart, and spleen were dehydrated with alcohol, embedded in paraffin wax, cut into 4-5 *μ*m thick sections, and stained with hematoxylin and eosin. Slides were examined in a light microscope at x40 magnification. The microscopic features of the organs of control and treated rats were compared. The study was carried out in the Veterinary Institute, Gannoruwa, Sri Lanka.

### 2.10. Statistical Analysis

All values are expressed as mean ± SEM. Comparisons between groups were performed using one-way analysis of variance (ANOVA) followed by Tukey's multiple comparison tests using SPSS statistical software. A *p* value of <0.05 is considered significant.

## 3. Results

### 3.1. Cytotoxic Property of Crude *n*-Hexane Extract of *A. malaccensis*

**Figure 1:**
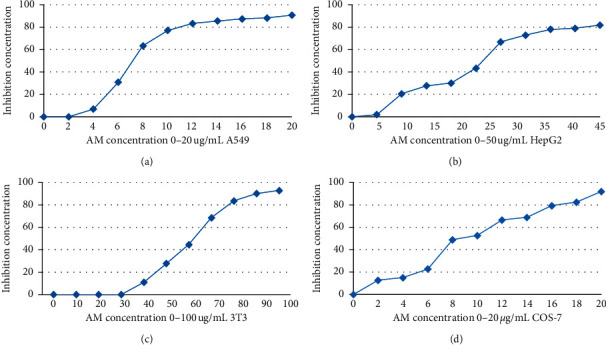
Dose response curve of *A. malaccensis* extract against different cell lines.

The cytotoxic property of *A. malaccensis n*-hexane extract was analyzed against A549, HepG2, 3T3, and COS-7 cells at different concentrations to determine the respective IC_50_ by MTT assay. The IC_50_ values of different concentrations of *A. malaccensis* extract are graphically represented in Figure 1. The IC_50_ values in respect of A549, HepG2, 3T3, and COS-7 cells were 7.25, 22.5, 62.75, and 8.25 *µ*g/mL, respectively. The extract showed 0% inhibition at concentrations 2, 1.4, 30, and 1.4 *µ*g/mL, for A549, HepG2, 3T3, and COS-7 cells, respectively, and these concentrations are considered as nontoxic.

### 3.2. Microscopic Features of Apoptosis and/or Necrosis

**Figure 2:**
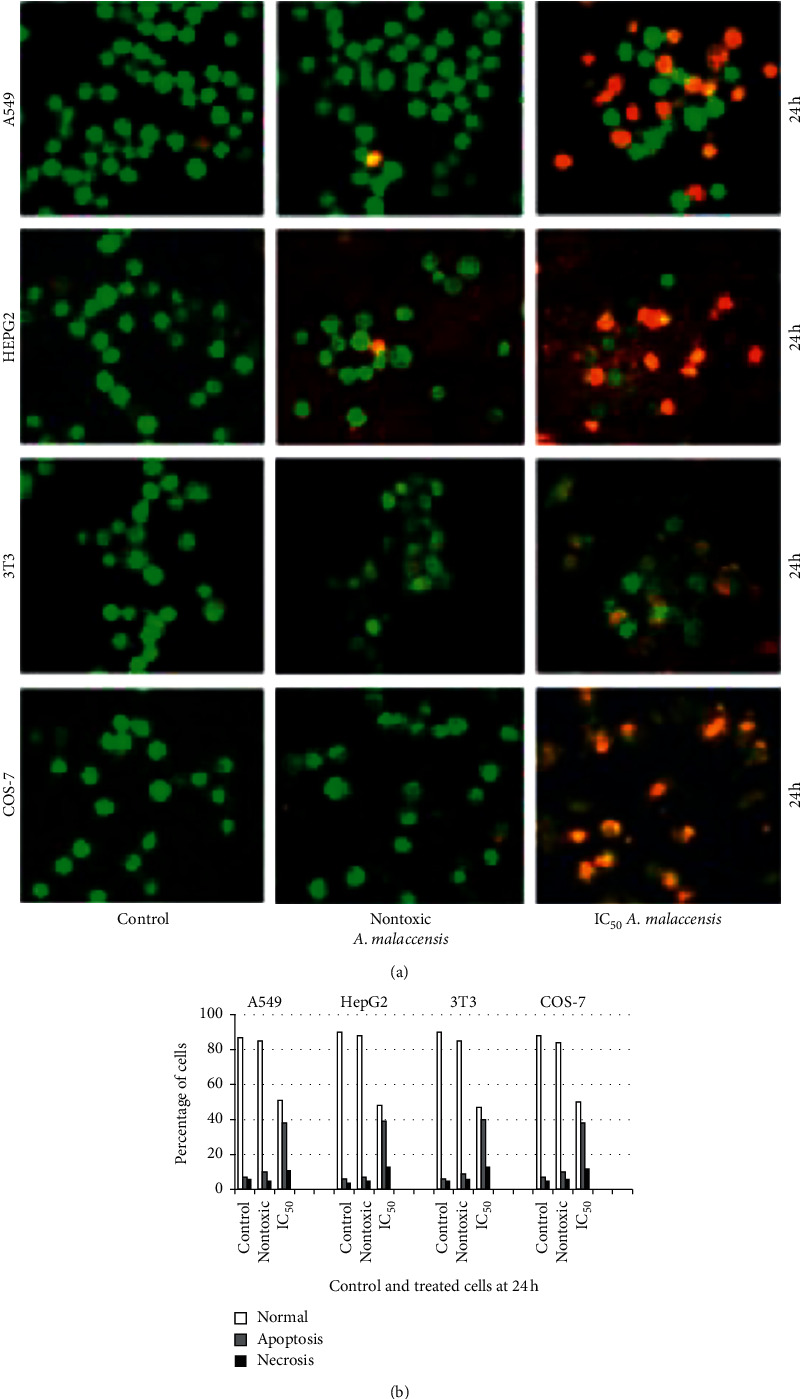
Assessment of apoptosis and necrosis. (a) Cells exposed to A. malaccensis extract and observed adopting AO/EB staining. Control cells are viable and fluoresce uniformly in green; cells in apoptosis fluoresce from yellow to orange; necrotic cells are swollen and fluoresce in bright red. (b) Percentage of normal, apoptotic, and necrotic cells. Data are expressed as the average of three independent observations.

Microscopic features of apoptosis were observed adopting AO/EB staining and are shown in Figure 2(a). In general, dead cells are permeable to EB and fluoresce red, whereas live cells are permeable to AO only and therefore fluoresce green. The viability and membrane integrity of the cells were determined based on the fluorescence pattern. The morphological changes observed in the treated cells were classified based on the fluorescence emission as follows: (i) viable cells having highly organized nuclei fluoresced green; (ii) early apoptotic cells which showed nuclear condensation fluoresced orange green; (iii) late apoptotic cells with the highly condensed chromatin or fragmented chromatin fluoresced orange to red; and (iv) necrotic cells fluoresced orange to red without chromatin fragmentation. Data on cells indicating apoptotic and necrotic morphologies, induced on treatment with the IC_50_ concentration and no toxicity concentrations of *A. malaccensis* for 24 h, and collected from manual counting are presented in Figure 2(b), which revealed that *A. malaccensis* at IC_50_ is efficient in bringing about early apoptosis (less than 50%), but little necrosis was produced. However, *A. malaccensis* at no toxicity concentration did not induce any remarkable apoptosis or necrosis (less than 10%) for the cell lines tested (Figure 2).

### 3.3. Microscopic Features of Nucleus

**Figure 3:**
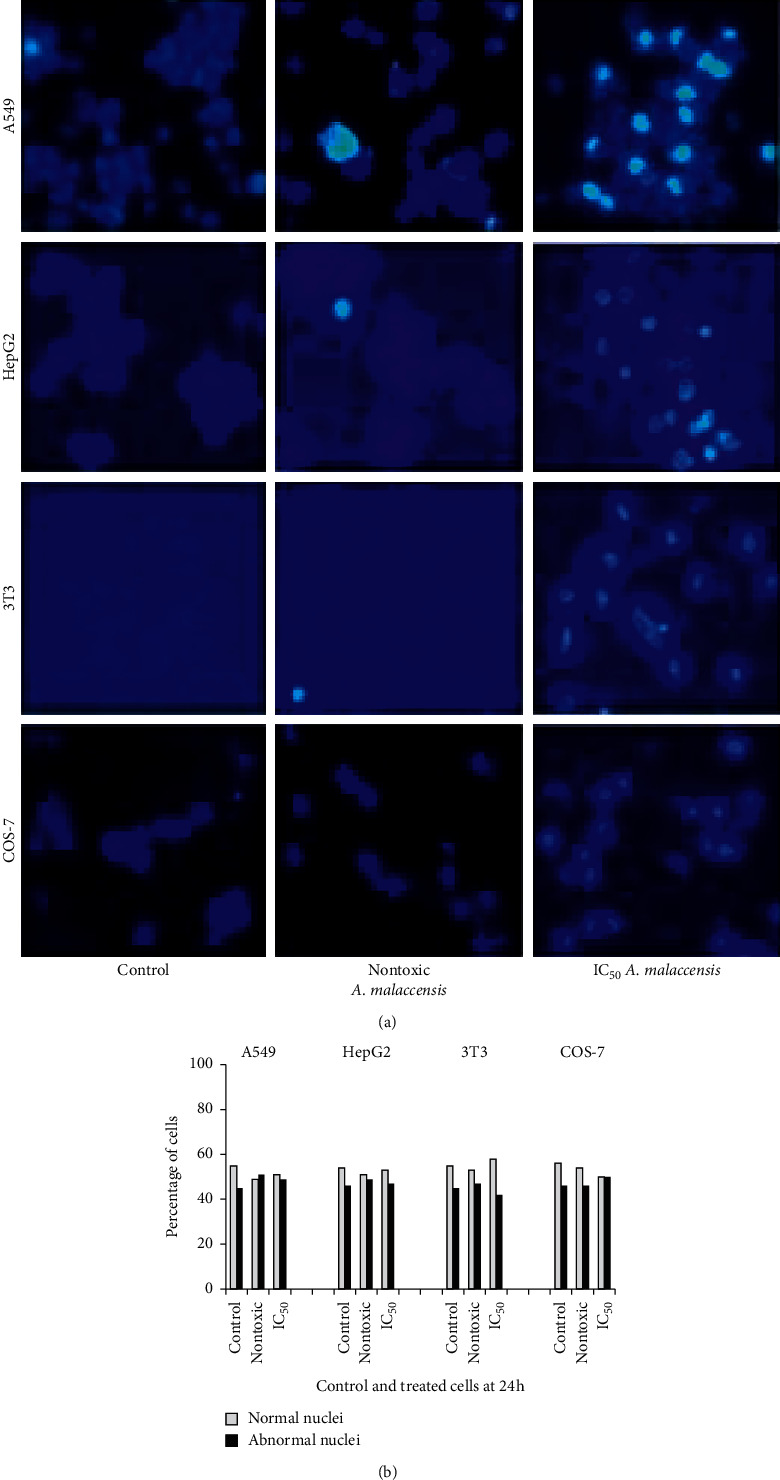
(a) Nuclear features of cells as revealed in Hoechst staining. (b) Percentage of cells with normal and abnormal nuclei. Data are expressed as average of three independent observations.

Hoechst 33528 staining was adopted to find the morphological changes in the nucleus as caused by treatment of IC_50_ and no toxicity concentrations of *A. malaccensis*, and the features are shown in Figure 3(a). Data collected from manual counting of cells with normal and abnormal nuclear features are shown in Figure 3(b). In the control cells, the nuclear chromatin was full while after treatment with the IC_50_ of the extract, changes such as chromatin marginalization, condensation, and fragmentation were noticed. These observations revealed that exposure of *A. malaccensis* to IC_50_ concentration alone led to chromatin fragmentation which is a characteristic feature of apoptosis. However, treatment with no toxicity concentration (IC_0_) of the extract indicated no difference with the control.

### 3.4. Identification of DNA Damage as Revealed in Comet Assay

**Figure 4:**
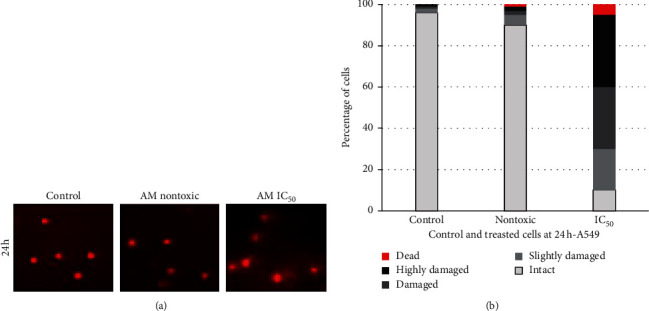
Assessment of DNA damage in A549 cells adopting comet assay. Data for the other cells are not shown since all showed similar trend. (a) DNA damage induced by *A. malaccensis* extract. Treated cells in which DNA has undergone strand breaks look like comets. (b) DNA damage as defined according to the DNA in the tail. The multiple parts of each column (from the bottom to the top): intact (0–20%), slightly damaged (20–40%), damaged (40–60%), highly damaged (60–80%), and dead (80–100%). Data are expressed as average of three independent experiments.

Comet assay was adopted to detect the cellular DNA lesions or genotoxicity. Comet assay is very sensitive of strand breaks in DNA. DNA damage was analyzed based on DNA tail size, shape, and migration pattern, and the data are shown in Figure 4(a). *A. malaccensis* at IC_50_ caused moderate damage to DNA. However, at the no toxicity concentration, there was only negligible damage to DNA. Since the tail length and density reflected the extent of strand breaks in DNA, the percentage of DNA in the tail provided a quantitative measure of DNA damage as shown in Figure 4(b).

### 3.5. Acute Oral Toxicity as Revealed in Animal Experiments

A single dose of 300 or 2000 mg/kg of *A. malaccensis* extract of the rhizome administered through oral route did not produce any mortality in rats during the 14-day observation period. Further, with regard to the behavioral patterns, rats treated 2000 mg of *A. malaccensis* extract were cleaning their face very frequently for the first 4 hours. However, after 4 h, the behavioral pattern was the same as in the control group.

### 3.6. Organ Weights of Tested Rats

Data on the organ weights between control and treated rats are shown in Table 1. The mean body weight of rats that received the two doses of *A. malaccensis* extract was not significantly different from the control group. The rats treated with the *A. malaccensis* extract at a dosage of 2000 mg/kg bw had liver weights significantly (*p* < 0.05) higher than control liver weight.

### 3.7. Biochemical Responses

Data on serum biochemical parameters of the rats are shown in Table 2. No significant difference (*p* > 0.05) in serum biochemical parameters was observed between rats treated *A. malaccensis* and control. Packed cell volume percentage (PCV%) was not significantly different between treated and control groups. Serum AST (u/L) and ALT (u/L) levels were not different between groups.

### 3.8. Hematological Responses

Data with regard to hematological parameters of rats are presented in Table 3. No significant difference (*p* > 0.05) was observed between treated and control groups.

### 3.9. Histopathological Observations of Organs

**Figure 5:**
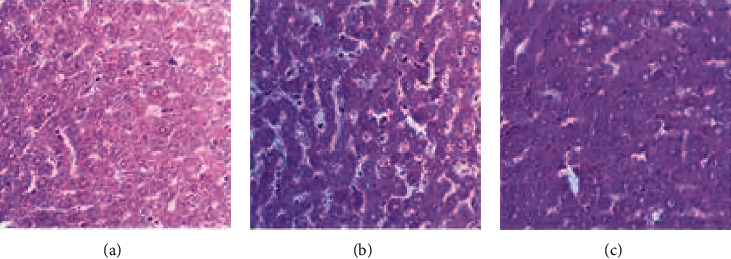
Photomicrographs of liver sections of rat. (a) Control. (b) *A. malaccensis* 300 mg/kg. (c) *A. malaccensis* 2000 mg/kg. Scale bar, 10 *µ*m.

The results of histopathological examination of liver sections of rats treated with olive oil (control) and different doses of the extract are shown in Figure 5. The liver tissue displayed normal hepatic cords and portal vein (Figure 5(a)) in the control groups. Liver tissue of rats administered the extract at 300 mg/kg did not show any alteration (Figure 5(b)), while the tissues of rats treated with 2000 mg/kg body weight showed mild congestion (Figure 5(c)).

**Figure 6:**
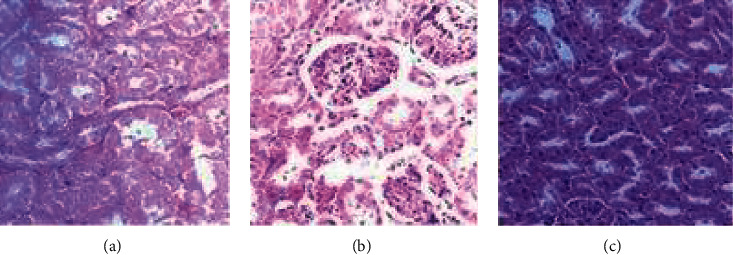
Photomicrographs of the kidney sections of rat. (a) Control. (b) *A. malaccensis* 300 mg/kg. (c) *A. malaccensis* 2000 mg/kg. Scale bar, 10 *µ*m.

The results of histopathological examination of kidney sections are shown in Figure 6. The kidney tissues presented intact tubules in control rats (Figure 6(a)). Rats administered the extract at 300 mg/kg body weight showed normal glomeruli and tubules (Figure 6(b)), while those in the 2000 mg/kg group showed glomeruli with mild lymphocytic infiltration (Figure 6(c)).

**Figure 7:**
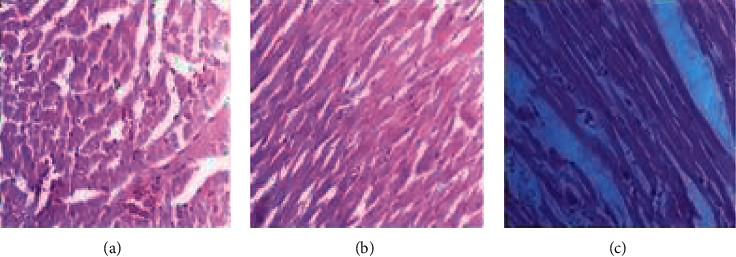
Photomicrographs of the heart sections of rat. (a) Control. (b) *A. malaccensis* 300 mg/kg. (c) *A. malaccensis* 2000 mg/kg. Scale bar, 10 *µ*m.

The histopathological examination of heart muscle sections of control rats revealed no pathological changes (Figure 7(a)). The rats in extract treatment at 300 mg/kg group also did not show any abnormality (Figure 7(b)), while those in the 2000 mg/kg group showed mild congestion (Figure 7(c)).

**Figure 8:**
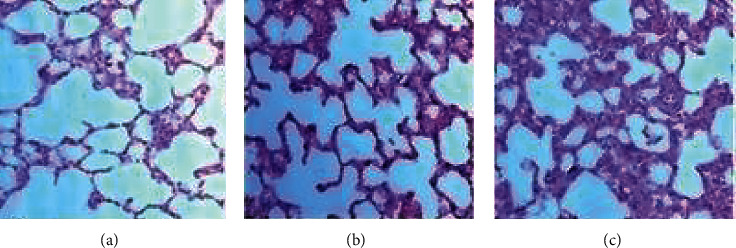
Photomicrographs of the lung sections of rat. (a) Control. (b) *A. malaccensis* 300 mg/kg. (c) *A. malaccensis* 2000 mg/kg. Scale bar, 10 *µ*m.

Lung sections of control rats are shown in Figure 8(a). The rats administered 300 and 2000 mg/kg extract did not show any perceivable microscopic changes (Figure 8(b)).

## 4. Discussion

Evaluation of toxicity, if any, of *A. malaccensis n*-hexane extract is the principal objective of this research since there is little scientific and clinical data on the effectiveness and the safety of *A malaccensis* rhizome extract. Single oral dose administration of 2000 mg/kg body weight of crude *A. malaccensis n*-hexane extract did not induce mortality or clinical signs of toxicity in rats throughout the observation period of 14 days. This indicated that the LD_50_ value of *A. malaccensis* extract is greater than 2000 mg/kg body weight. Although all treated rats showed normal behavior during 24 h, some animals showed sign of mild distress during the first 4 h and drank too much water during the first 3 h after oral administration of the extract. According to Teo et al. [23], changes in the body weight can be considered as markers of adverse effects upon oral administration of drugs and chemicals. More than 10% body weight loss from the initial body weight is considered as significant (*p* > 0.05 %) [24]. In our study, none of the animals showed such reduction in body weight, but their body weights increased during the observation period indicating that the plant extract did not produce any adverse effect on the body weight of rats in terms of acute oral toxicity. Similar observations were reported by Karunarathne et al. [13] when they administered 2000 mg/kg body weight of crude *Alpinia galanga n*-hexane extract on rats, which is a plant extract of the same genus having 1'ACA as the major chemical compound. Furthermore, no change of organs was observed in the treated rat with the exception being the liver of rats treated with 2000 mg/kg bw day of *A. malaccensis* crude extract. Assessment of liver and kidney function is a very vital index in evaluating the toxicity of the plant extracts. The serum urea, creatinine, and liver enzyme (ALT, AST, and ALP) concentrations of the blood were the indices used to evaluate the function of kidney [25]. The results in respect of BUN, creatinine, ALT, and AST after 14 days of oral administration were not significantly different from the control group and revealed that there was no abnormality of the kidney for all the treated rats. Hematopoietic system is one of the most susceptible targets of toxic compounds, especially the bone marrow where the production of red blood cell occurs [26]. Acute administration of the plant extract did not cause any significant changes in the hematological profile of rats which received the *A. malaccensis* extract at different doses, suggesting that the plant extract does not affect the hematopoietic system. The treatment did not bring about any serious histopathological change in the liver, kidney, heart, and spleen. Therefore, it is to be inferred that the plant extract, at least up to 2000 mg/kg, is not toxic in respect to acute oral administration. However, before a final decision, subacute and chronic toxicity testing of *A. malaccensis* will be highly relevant.

*In vitro* studies are equally important in deciphering if a material is toxic or not. Different cell lines might exhibit different sensitivities towards a compound if it is cytotoxic. Therefore, use of more than one cell line is considered necessary in the detection of cytotoxic effects [27]. So, we found the IC_50_ values of *A. malaccensis n*-hexane extract for A549, HepG2, 3T3, and COS-7 cell lines which were 7.25, 22.5, 62.75, and 8.25 *µ*g/mL, respectively, and the zero-toxic concentration (IC_0_) was 2, 1.4, 30, and 1.4 *µ*g/mL, respectively. This is important since so far no toxicity data have been reported in literature for *A. malaccensis* [28].

Further, the mode of cell death, whether apoptosis or necrosis, induced by the plant extract was assessed. Double staining of AO/EB for nontoxic concentration of *A. malaccensis* showed nuclear and morphological changes of apoptosis only in less than 10% of the cells. However, more than 10% apoptosis was observed for cells treated the IC_50_ concentration of *A. malaccensis* extract. Similar observations were made for Hoechst staining. However, comet assay revealed that 7.25 *µ*g/mL of *A. malaccensis* IC_50_ induced severe damage to DNA of A549 cell. In contrast, plant extracts at non-cytotoxic concentration(s) did not induce DNA damage in A549 cell. Thus, our results clearly demonstrated that *A. malaccensis* at nontoxic doses did not exert genotoxicity in A549 cells. In fact, the extract at these nontoxic concentrations could be used as preservative of food stored for human consumption without any adverse effect on human health. Thus, considering the nontoxic concentrations, acceptable daily intake (ADI) of the extract could be approximately calculated using NOAEC (no-observed adverse effect concentration) divided by uncertainty factor (usually10 to extrapolate from animal to human and 10 for interindividual differences in sensitivity). NOAEC is defined as the concentration at which there is no risk (nontoxic level) [29]. The approximate ADI value for *A. malaccensis n*-hexane extract is 55.41 mg/day (data not shown). However, bioavailability of the extract(s), animal data, or PBPK (physiologically based biokinetic model) is necessary to derive an appropriate ADI for humans. Therefore, we recommend further investigations on subchronic and chronic toxicity testing to find the reliability of the estimated ADI of the plant extract when used as food preservative and/or therapeutic. Put together, the most nonpolar (*n*-hexane) extract of *A. malaccensis* offers great potential as a food preservative as well as therapeutic which could be further established through clearly directed studies.

## 5. Conclusion

The oral toxicity studies in the rat model revealed that *A. malaccensis n*-hexane extract at a single dose of 2000 mg/kg body weight did not produce any serious adverse effect with respect to general behavior, body weight, feed intake, biochemical parameters, and organ histology. In addition, cytotoxicity analysis showed that nontoxic concentration of *A. malaccensis* (2, 1.4, 30, and 1.4 *µ*g/mL, respectively) does not affect cell viability or the DNA. Therefore, these concentrations may be practiced for human consumption without any adverse health effect. However, we recommend subchronic and chronic toxicity tests to further exonerate the adverse effects by repeated administration of *A. malaccensis.* Further, it is possible that higher doses of *A. malaccensis* extract would potentially damage DNA, and therefore, we recommend further research towards standardization of *A. malaccensis* as preservative of food meant for human consumption and/or as a therapeutic.

## Figures and Tables

**Table 1:** Organ weights (g) of female rats.

Organ	Weight in grams	*A. malaccensis* extract (mg/kg)	
	Control	300	2000
Liver	6.05 ± 0.40^b^	6.09 ± 0.42^b^	7.12 ± 0.18^a^
Left kidney	0.56 ± 0.06	0.53 ± 0.01	0.67 ± 0.06
Right kidney	0.57 ± 0.09	0.54 ± 0.38	0.66 ± 0.03
Lungs	0.98 ± 0.08	1.00 ± 0.05	1.16 ± 0.05
Heart	0.68 ± 0.04	0.68 ± 0.04	0.75 ± 0.04
Spleen	0.44 ± 0.04	0.42 ± 0.02	0.50 ± 0.02

Data with different lower case letters are significantly different (*p* < 0.05).

**Table 2:** Serum biochemical parameters of female rats.

Biochemical parameter	*A. malaccensis* extract (mg/kg)		
	Control	300	2000
PCV (%)	43.00 ± 1.41^*∗*^	43.66 ± 0.47	43.66 ± 0.47
ALT(u/L)	34.33 ± 2.50	31.06 ± 1.55	28.40 ± 5.39
AST (u/L)	117.00 ± 2.08	117.00 ± 0.00	119.65 ± 1.05
BUN (urea/mg)	15.53 ± 2.69	15.23 ± 2.20	16.03 ± 1.10
Creatinine (mg/dL)	0.63 ± 0.09	0.93 ± 0.09	0.87 ± 0.12

^*∗*^Results are expressed as mean ± SEM of 3 samples.

**Table 3:** Serum hematological values of female rats.

Hematological parameter	Control	*A. malaccensis* extract	
		300 mg/kg	2000 mg/kg
Hb (g/dL)	12.60 ± 0.14^*∗*^	14.00 ± 0.84	13.13 ± 0.71
WBC (×103 mm)	9.00 ± 0.71	11.20 ± 1.57	8.66 ± 0.30
RBC (106/L)	6.78 ± 0.89	7.20 ± 0.21	6.53 ± 0.41
Neutrophils (109/L)	16.33 ± 0.76	15.66 ± 5.43	13.00 ± 0.81
Lymphocytes (109/L)	81.33 ± 8.99	82.66 ± 5.55	83.66 ± 0.41
Erythrocytes (109/L)	0.33 ± 0.47	0.33 ± 0.47	1.00 ± 0.81
Monocytes (109/L)	2.00 ± 0.82	1.33 ± 0.47	2.33 ± 0.47

^*∗*^Results are expressed as mean ± SEM of 3 samples.

## Data Availability

The data of organ weights, serum biochemical parameters, serum hematological values of female rats, *in vitro* inhibition concentration of *A*. *malaccensis* on different cell lines, morphological assessment on apoptosis and necrosis, nuclear features of cells, assessment of DNA damage, and photomicrographs of internal organ sections used to support the findings of this study are included within the article.

## References

[1] World Health Organization (2002). *WHO Traditional Medicine Strategy 2002–2005*.

[2] Karimi A., Majlesi M., Rafieian-Kopaei M. (2015). Herbal versus synthetic drugs; beliefs and facts. *Journal of Nephropharmacology*.

[3] Ertekin V., Selimoğlu M. A., Altinkaynak S. (2005). A combination of unusual presentations of *Datura stramonium* intoxication in a child: rhabdomyolysis and fulminant hepatitius. *The Journal of Emergency Medicine*.

[4] Koduru S., Grierson D. S., Afolayan A. J. (2006). Antimicrobial activity of *Solanum aculeastrum*. *Pharmaceutical Biology*.

[5] Sirikantaramas S., Yamazaki M., Saito K. (2008). Mechanisms of resistance to self-produced toxic secondary metabolites in plants. *Phytochemistry Reviews*.

[6] Raj G., Pradeep D. P., Yusufali C., Dan M., Baby S. (2012). Chemical profiles of volatiles in four Alpinia species from Kerala, South India. *Journal of Essential Oil Research*.

[7] Bhuiyan M. N. I., Chowdhury J. U., Begum J., Nandi N. C. (2010). Essential oils analysis of the rhizomes of *Alpinia conchigera Griff*. and leaves of A*lpinia malaccensis* (Burm.f.) Roscoe from Bangladesh. *African Journal Plant Science*.

[8] Somarathna T., Fernando W. M. A. D. B., Ranaweera K. K. D. S., Premakumara G. A. S., Abeysinghe T., Weerakkody N. S. (2018). Antimicrobial activity and phytochemical screening of *Alpinia malaccensis* (Ran-kiriya) against food-borne bacteria. *Journal of Applied Microbiology*.

[9] Somarathna T., Premakumara G. A. S., Akbarsha M. D., Balamuthu K. (2017). In vitro antibacterial activity of selected underutilized plants and cytotoxic property of *Terminalia catappa*. *International Journal of Pharmacy and Pharmaceutical Sciences*.

[10] Primahana G., Ernawati T., Dewi N. L. P., Dwiyatmi I. D., Darmawan A., Hanafi M. (2015). Synthesis of 2-allylphenyl cinnamate and brine shrimp lethality test activity evaluation. *Procedia Chemistry*.

[11] Thu N. B., Trung T. N., Khoi N. M (2010). Articles: screening of Vietnamese medicinal plants for cytotoxic activity. *Natural Product Sciences*.

[12] Janssen A., Scheffer J., Svendsen A. (1987). Antimicrobial activity of essential oils: a 1976–1986 literature review. Aspects of the test methods. *Planta Medica*.

[13] Karunarathne P. U. H. S., Thammitiyagodage M. G., Weerakkody N. S. (2018). Safety evaluation of galangal (*Alpinia galanga*) extract for therapeutic use as an antimicrobial agent. *International Journal of Pharmacutical Science. and Research*.

[14] Chan E., Lim Y., Omar M. (2007). Antioxidant and antibacterial activity of leaves of Etlingera species (Zingiberaceae) in Peninsular Malaysia. *Food Chemistry*.

[15] Dassanayake M., Forsberg R. (1983). *A Revised Handbook to the Flora of Ceylon*.

[16] Gayathri L., Dhivya R., Dhanasekaran D., Periasamy V. S., Alshatwi A. A., Akbarsha M. A. (2015). Hepatotoxic effect of ochratoxin A and citrinin, alone and in combination, and protective effect of vitamin E: in vitro study in HepG2 cell. *Food and Chemical Toxicology*.

[17] Mosmann T. (1983). Rapid colorimetric assay for cellular growth and survival: application to proliferation and cytotoxicity assays. *Journal of Immunological Methods*.

[18] Nemati F., Dehpouri A., Eslami B., Mahdavi V., Mirzanejad S. (2013). Cytotoxic properties of some medicinal plant extracts from mazandaran, Iran. *Iranian Red Crescent Medical Journal*.

[19] Spector D. L., Goldman R. D. (1998). *Cell: A Laboratory Manual. Culture and Biochemical Analysis of Cells*.

[20] Latt S. A., Stetten G., Juergens L. A., Willard H. F., Scher C. D. (1975). Recent developments in the detection of deoxyribonucleic acid synthesis by 33258 Hoechst fluorescence. *Journal of Histochemistry & Cytochemistry*.

[21] Singh N. P., McCoy M. T., Tice R. R., Schneider E. L. (1988). A simple technique for quantitation of low levels of DNA damage in individual cells. *Experimental Cell Research*.

[22] Saboudry M. (1993). *A Breeding and Care of laboratory Animals*.

[23] Teo S. K., Stirling D. I., Thomas S. D., Hoberman A. M., Christian M. S., Khetani V. D. (2002). The perinatal and postnatal toxicity of D-methylphenidate and D,L-methylphenidate in rats. *Reproductive Toxicology*.

[24] Williamson D., Bray G., Ryan D. (2015). Is 5% weight loss a satisfactory criterion to define clinically significant weight loss?. *Obesity (Silver Spring, Md.)*.

[25] Chavda R., Vadalia K. R., Gokani R. (2010). Hepatoprotective and antioxidant activity of root bark of *Calotropis procera* R.Br (asclepediaceae). *International Journal of Pharmacology*.

[26] Kifayatullah M., Mustafa M. S., Sengupta P., Sarker M. M. R., Das A., Das S. K. (2015). Evaluation of the acute and sub-acute toxicity of the ethanolic extract of *Pericampylus glaucus* (Lam.) Merr. in BALB/c mice. *Journal of Acute Disease*.

[27] Khairunnisa K., Karthik D. (2014). Evaluation of in-vitro apoptosis induction, cytotoxic activity of *Hymenodictyon excelsum* (roxb) wall in dalton’s lymphoma ascites (DLA) and lung fibroblast-mouse l929 cell lines. *Journal of Applied Pharmaceutical Science*.

[28] Sahoo S., Singh S., Nayak S. (2014). Chemical composition, antioxidant and antimicrobial activity of essential oil and extract of *Alpinia malaccensis roscoe* (Zingiberaceae). *International Journal of Pharmacy and Pharmaceutical Sciences*.

[29] Food and Agriculture Organization (FAO) (2009). Principles and Methods for the Risk Assessment of Chemicals in Food. *Chapter 7. Risk Characterization*.

